# Birthweights and Down syndrome in neonates that were delivered after frozen‐thawed embryo transfer: The 2007‐2012 Japan Society of Obstetrics and Gynecology National Registry data in Japan

**DOI:** 10.1002/rmb2.12033

**Published:** 2017-04-10

**Authors:** Kenji Yamatoya, Kazuki Saito, Takakazu Saito, Woojin Kang, Akihiro Nakamura, Mami Miyado, Natsuko Kawano, Yoshitaka Miyamoto, Akihiro Umezawa, Kenji Miyado, Hidekazu Saito

**Affiliations:** ^1^ Department of Perinatal Medicine and Maternal Care National Center for Child Health and Development Tokyo Japan; ^2^ Department of Reproductive Biology National Research Institute for Child Health and Development Tokyo Japan; ^3^ Department of Molecular Endocrinology National Research Institute for Child Health and Development Tokyo Japan; ^4^ Department of Life Sciences School of Agriculture Meiji University Kawasaki Japan

**Keywords:** assisted reproductive technology, birthweight, chromosomal abnormalities, Down syndrome, frozen‐thawed embryo transfer

## Abstract

**Aim:**

To evaluate the use of frozen embryos on the outcome of assisted reproductive technology (ART), a retrospective study of the Japanese Assisted Reproductive Technology Registry data during the years 2007‐2012 was conducted.

**Methods:**

A total of 124 946 singleton neonates who reached term gestation following ART from 2007‐2012, with 80 660 achieved through frozen‐thawed embryo transfer (ET) and 44 286 being achieved through fresh ET, were analyzed for their birthweights and chromosomal abnormalities.

**Results:**

The birthweight of the neonates from the frozen‐thawed ETs was significantly higher than that of those from the fresh ETs throughout all the study years. The frequency of Down syndrome was 0.17% for the fresh ETs and 0.13% for the frozen‐thawed ETs in the period 2007‐2012. This study showed that frozen‐thawed ETs result in a constant increase of the average birthweight between 37 and 41 weeks gestational age and lower frequencies of Down syndrome.

**Conclusion:**

Frozen‐thawed ETs were comparable to the fresh ET method, with the exceptions of higher birthweights and a lower frequency of Down syndrome in the neonates that were born from frozen‐thawed ET. The increase in birthweights was not proportional to the gestational ages. This cannot be explained with any well‐known mechanism. The frequency of chromosomal abnormalities needs detailed data for analysis.

## 1 Introduction

Assisted reproductive technology (ART) is now a widely accepted medical treatment for infertility. The number of children who were born to couples undergoing ART has been increasing steadily and was reported to have reached an estimated 5 million in 2012 globally.^1^ Currently, the infertility criteria include endocrine disturbance, hypogonadism, tubal damage, malignant diseases, urogenital infection, and other unexplained cases. Likewise, either female or male factors, or a combination of both, can contribute to the symptoms. In order to handle varying cases, multiple methods, such as ovarian stimulation and luteal phase support, are being developed and improved constantly. Notably, eggs and sperm can be handled by in vitro methods, such as in vitro fertilization (IVF) and intracytoplasmic sperm injection, whereby embryos are often cryopreserved in preparation for repeat treatment. In order to explore the safety of ART treatments for neonatal outcomes, large‐scale studies based on the data in national registry systems have been undertaken in several countries.^2^, ^3^, ^4^, ^5^, ^6^, ^7^

As pregnancies that are achieved by ART treatments are associated with a higher incidence of a low birthweight,^2^, ^7^, ^8^, ^9^, ^10^, ^11^ repeat ART treatments are considered to be one of the risks that are attributed to preterm birth. However, it has been reported previously that the neonatal birthweight might be higher with the frozen‐thawed embryo transfer (ET) method than that with the fresh ET method.^2^, ^12^ However, the various factors that affect the birthweight remain largely unknown at present.

In one study, the birthweight of the neonates who were born by way of ART treatments was analyzed, based on 2 years of data that had been retrieved from the Japanese national registry of ART in 2007 and 2008.^2^ It was demonstrated that the birthweight after frozen‐thawed ET was significantly higher, when compared with fresh ET, as well as all other, Japanese births.^2^ In other words, the risk of a low neonatal birthweight with frozen‐thawed ET was significantly lower, when compared with that of the fresh ET method. Also, the risk of a low birthweight with shorter in vitro culture times was significantly higher than that with the longer culture times with the fresh ET method.

In order to further evaluate the implications of frozen‐thawed ET on birthweights, the authors assessed continuous 6 year data that were included in the Japanese National Registry of ART from the years 2007 to 2012. The annual changes in the numbers and birthweights of the neonates from the fresh ETs and the frozen‐thawed ETs from 2011‐2012 also were analyzed in order to assess the effect of the Great East Japan Earthquake.

## 2 Materials and Methods

### 2.1 Assisted reproductive technology*‐*treated patients who were recorded in the registry system

The Japan Society of Obstetrics and Gynecology (JSOG) provided the data that were used for this study. The number of institutes that was recorded in the Japanese national assisted reproduction registry were 549, 548, 548, 552, 551, and 555 institutes in 2007, 2008, 2009, 2010, 2011, and 2012, respectively. These institutes were registered with the JSOG online registration system and were required to record the patients’ ages, locations (prefecture, institution), subvention in ART treatment, protocols used in the treatment, and obstetrical outcomes. A summary of these registered data is released annually on the JSOG home page. For the present study, the patients’ information and the outcomes of their treatments were extracted from the JSOG database for the period between 2007 and 2012. In total, there were 80 660 cycles of frozen‐thawed ET and 44 286 cycles of fresh ET singleton births after incomplete data were excluded from the analysis.

### 2.2 Assisted reproductive technology treatments that were included in the registry data

The ET methods were categorized into two groups: frozen‐thawed or fresh ET cycle. In order to exclude the premature birth complications, the gestational ages of 37‐41 weeks were extracted. Multiple births, neonatal mortalities, and obvious transcription errors were excluded. The birthweights of the neonates who resulted from the frozen‐thawed and fresh ET cycles were compared. The cleavage stage of the embryos at ET and the date of cryopreservation could not be considered because they were not included in the registry data.

### 2.3 Statistical analysis

The statistical comparisons were made by using Welch's *t* test or Fisher's exact test. Statistical significance was defined as *P*<.05. The odds ratios (ORs) with 95% confidence intervals (CIs) were estimated.

### 2.4 Ethical considerations

The Registration and Research Subcommittee of the JSOG Ethics Committee approved this study and the dataset that was provided was in accordance with the guidelines that had been set forth by the Committee.

## 3 Results

### 3.1 Increased rate of frozen‐thawed cycles

The obtained data from 12 495 (2007), 15 734 (2008), 19 797 (2009), 22 089 (2010), 25 058 (2011), and 29 773 (2012) singleton pregnancies were analyzed (Figure S1A). Based on these data, 124 946 cycles of singleton births at term gestation were investigated. As shown in Figure S1B, the cycles of ART treatments with frozen‐thawed ET were lower than that of fresh ET in 2007 (6042 cycles [48.4%] vs 6453 cycles [51.6%]). However, this trend was reversed in 2008 (9109 cycles with frozen‐thawed ET [57.9%] vs 6625 cycles with fresh ET [42.1%]). Thereafter, the number of cycles with frozen‐thawed ET tended to further increase (62.0%, 65.6%, 68.6%, and 72.4%) in 2009, 2010, 2011, and 2012, respectively. For the 6 year period between 2007 and 2012, there were 80 660 cycles of frozen‐thawed ET (64.6%) and 44 286 cycles of fresh ET (35.4%).

### 3.2 Neonatal birthweight after frozen‐thawed embryo transfer

**Figure 1:**
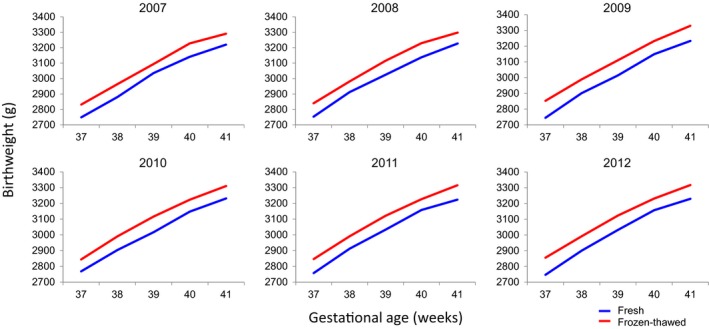
Trends in the neonatal birthweights following term gestation. The patient outcomes and treatment data were extracted from the Japan Society of Obstetrics and Gynecology database during the period from 2007 to 2012. The data are expressed as the mean values

In order to clarify the relationship between the frozen‐thawed ET cycles and the birthweights of the neonates, the birthweights were compared between those from the frozen‐thawed ET, compared to the fresh ET, cycles (Figure 1). As reported previously, the mean birthweight of the neonates tended to be lower with the fresh ET cycles than that with the frozen‐thawed ET cycles for the years 2007 and 2008.^2^ In order to verify the differences in the birthweights between these two groups, the birthweights from 2009 to 2012 were examined. The results showed that the birthweights after frozen‐thawed ET was ~90 g heavier, compared to the birthweights with fresh ET, for any of the gestational weeks in 2007 and 2008. This trend was also apparent in 2009, 2010, 2011, and 2012 (Figure 1). These results suggest that the neonatal birthweight tends to be increased with the frozen‐thawed ET cycles. Concerning the risk of macrosomia, the rate of neonates who were >4500 g was estimated between the frozen‐thawed and the fresh embryos by using the latest 2012 registry data. The result showed no significant difference between these two groups (OR=3.1, 95% CI=0.98‐9.5; *P*=.055).

### 3.3 Birthweights in 2011 and 2012

Since the Great East Japan Earthquake occurred in 2011, it was considered that environmental and emotional factors could have influenced the oocyte quality and that the use of frozen‐thawed embryos might have been able to overcome this influence. Therefore, the national registry data were analyzed from 2011 (Figure S2). The gestational age was limited to 39 weeks and the birthweight is displayed as histograms at 50 g intervals. Figure S2A shows the number of neonates who exhibited twin peaks from 3000 to 3050 g and from 3150 to 3200 g after the frozen‐thawed ET cycles and from 2950 to 3000 g and from 3050 to 3100 g after the fresh ET cycles in 2011. Furthermore, when the frequency of the neonates from the fresh ET cycles was compared to that of the neonates from the frozen‐thawed ET cycles, the difference in the two types of cycles was more evident (Figure S2B).

In order to further investigate the registry data, additional histograms were used to depict the data that were registered in 2012. Figure S3A shows the number of neonates who exhibited a single peak from 3100 to 3150 g after the frozen‐thawed ET cycles. This peak value was located between the two peaks in the histogram from the 2011 data (Figure S2A). Figure S3A shows the number of neonates who displayed a single peak from 2950 to 3000 g in the case of the fresh ET cycles and this peak value was consistent with the lower peak value from 2011. Furthermore, when the frequency of the neonates from the fresh ET cycles was compared to that of the neonates from the frozen‐thawed ET cycles, the difference in the two types of cycles was more evident (Figure S3B).

### 3.4 Regional distribution of the neonates

**Figure 2:**
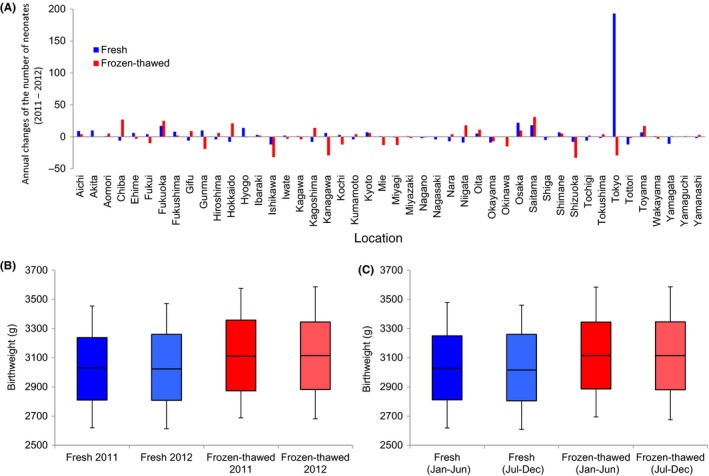
Comparisons of the number of neonates and birthweights in 2011 and 2012. A, Changes in the number of neonates from 2011 to 2012. B, The birthweights of the neonates in 2011 and 2012. The *P*‐values between the fresh and the frozen‐thawed groups were <.0001. C, The birthweights of the neonates in the half‐years of 2012. The *P*‐value between the January‐June and July‐December fresh groups was 0.66. The *P*‐value between the January‐June and July‐December frozen‐thawed groups was 0.79. For each group, the line in the middle of the box represents the median. The lower and the upper edges of the box are the 25th and 75th percentile, respectively. The lower and the upper whiskers represent the 10th and 90th percentile, respectively

In order to investigate the relationship between the birthweight and the ET cycle, the registry data, limited to 39 weeks of gestational age in 2011 and 2012, were further analyzed. As Japan consists of 47 prefectures, the distribution of the neonates is shown at the prefecture level (Figure 2A). When the number of neonates was compared between these 2 years, the number of neonates who were born from the fresh ET cycles increased in 2012, compared to that observed in 2011, strikingly in Tokyo (Figure 2A). Furthermore, the birthweight was significantly lower in the neonates who were born from the fresh ET cycles than that of the neonates who were born from the frozen‐thawed ET cycles (*P*=1.2×10^−20^) in 2011 (Figure 2B). Similarly, the birthweight of the neonates who were born from the fresh ET cycles was significantly lower than that of the neonates who were born from the frozen‐thawed ET cycles in 2012 (*P*=5.4×10^−26^). However, the birthweight of the neonates who were born from the frozen‐thawed cycles was comparable between 2011 and 2012, even for the neonates who were born from the fresh ET cycles between 2011 and 2012 (Figure 2B). To further examine any seasonal differences, the registry data that were obtained in 2012 was divided into two groups; that is, from January to June and from July to December. The birthweight was significantly lower in the neonates from the fresh ET cycles than in those from the frozen‐thawed ET cycles from January to June in 2012 (*P*=1.2×10^−12^) and from July to December in 2012 (*P*=6.3×10^–14^) (Figure 2C). In contrast, the birthweight was comparable between the neonates from the frozen‐thawed cycles between these two seasons in 2012 and even between the neonates from the fresh ET cycles (Figure 2C). This result suggested that the Great East Japan Earthquake had no effect on the birthweight of neonates from ET cycles.

### 3.5 Frequency of Down syndrome

**Figure 3:**
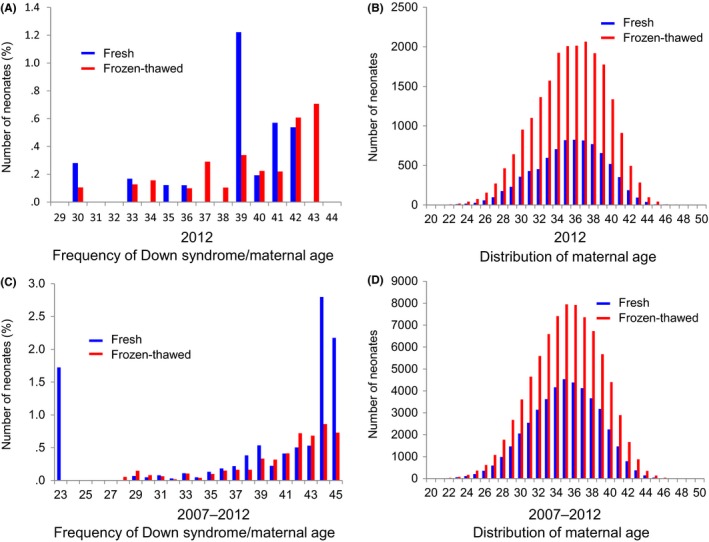
Frequency of Down syndrome in the neonates from the frozen‐thawed, compared to the fresh embryo transfer, cycles. A, The number of neonates with Down syndrome. The data were retrieved from the Japan Society of Obstetrics and Gynecology (JSOG) database in 2012. B, The distribution in maternal age of the patients who underwent the frozen‐thawed, compared to the fresh embryo transfer, cycles in 2012. C, The number of neonates with Down syndrome. The data were retrieved from the JSOG database during the period from 2007 to 2012. D, The distribution in the maternal age of the patients who underwent the frozen‐thawed, compared to the fresh embryo transfer, cycles during the period from 2007 to 2012

To a great extent, chromosomal abnormalities, known as aneuploidy, are responsible for failures with the implantation of IVF embryos.^13^, ^14^ Notably, Down syndrome is the most common chromosomal disorder. Therefore, the frequency of chromosomal abnormalities was examined between frozen‐thawed and fresh ET cycles in 2012 (Figure 3A, Table 1) and the 6 year period between 2007 and 2012 (Figure 3C, Table 1). It was observed that the frequency of Down syndrome and entire chromosomal abnormalities was comparable between the neonates who were born from the frozen‐thawed ET cycles, compared to the fresh ET cycles, in 2012 (Figure 3A, Table 1). The entire chromosomal abnormalities in the 6 year period between 2007 and 2012 were also similar between the neonates who were born from the frozen‐thawed ET cycles, compared to the fresh ET cycles (Table 1). However, the frequency of Down syndrome in the 6 year period between 2007 and 2012 was significantly higher in the neonates who were born from the fresh ETs than in the neonates who were born from the frozen‐thawed ETs (Figure 3C, Table 1). The average age of the mothers of a neonate with Down syndrome was 38 years for both the fresh ETs and the frozen‐thawed ETs (Figure 3A). The average age of the mothers of a neonate with Down syndrome who had undergone a fresh ET was 38 years and for those who had undergone a frozen‐thawed ET, it was 37.9 years (Figure 3C). The average age of the mothers of all the neonates in Figure 3B was 35.413 years for the fresh ETs and 35.362 years for the frozen‐thawed ETs. The average age of the mothers of all the neonates in Figure 3D was 34.947 years for the fresh ETs and 35.065 years for the frozen‐thawed ETs. The maternal age was comparable between the patients undergoing either method (Figure 3B,D).

**Table 1:** Frequencies of chromosomal abnormalities. Chromosomal abnormalities in 2012. and chromosomal abnormalities during the period from 2007 to 2012

Chromosomal abnormality	Chromosomal abnormalities in 2012 (%)					Chromosomal abnormalities from 2007‐2012 (%)				
	Fresh (n=8230)	Frozen‐thawed (n=21 543)	OR	95% CI	*P*	Fresh (n=44 286)	Frozen‐thawed (n=80 660)	OR	95% CI	*P*
Beckwith–Wiedemann syndrome	0 (0.00)	2 (0.01)	–	–	–	0 (0.00)	4 (0.00)	–	–	–
Prader–Willi syndrome	0 (0.00)	0 (0.00)	–	–	–	1 (0.00)	1 (0.00)	0.55	0.6–5.26	1.00
5p‐	1 (0.01)	0 (0.00)	–	–	–	1 (0.00)	0 (0.00)	–	–	–
Trisomy 13	0 (0.00)	1 (0.00)	–	–	–	0 (0.00)	2 (0.00)	–	–	–
Trisomy 18	0 (0.00)	3 (0.00)	–	–	–	3 (0.01)	9 (0.01)	1.65	0.48–5.36	.56
Trisomy 21	16 (0.19)	32 (0.15)	0.76	0.42–1.38	.42	77 (0.17)	101 (0.13)	0.72	0.54–0.97	.03
XYY	0 (0.00)	1 (0.00)	–	–	–	1 (0.00)	1 (0.00)	0.55	0.06–5.26	1.00
XXY	0 (0.00)	1 (0.00)	–	–	–	1 (0.00)	5 (0.01)	2.75	0.43–17.72	.43
xxx	0 (0.00)	0 (0.00)	–	–	–	1 (0.00)	0 (0.00)	–	–	–
Others (deletions, insertions, inversions, translocations)	0 (0.00)	1 (0.00)	–	–	–	4 (0.01)	8 (0.01)	1.10	0.35–3.43	1.00
Total	17 (0.21)	41 (0.19)	0.92	0.53–1.61	.77	89 (0.20)	131 (0.16)	0.81	0.62–1.06	.12

CI, confidence interval; OR, odds ratio.

## 4 Discussion

In the present study, a retrospective analysis was conducted of the clinical data on 124 946 singleton neonates who were delivered at term following ART treatment. The data were retrieved from the national ART registry of Japan for the years 2007‐2012. It was observed that the number of treatments with the frozen‐thawed ET method has steadily increased between 2007 and 2012 in Japan and prevailed in comparison to that of the fresh ET method. Despite the gestational age, the birthweight increased in the neonates from the frozen‐thawed ETs, compared with that of those from the fresh ETs throughout the 6 year period that was analyzed. The frequency of Down syndrome decreased in the neonates from the frozen‐thawed ETs, compared with those from the fresh ETs, in the 6 year period that was examined.

### 4.1 Trends in the usage of frozen‐thawed embryo transfers

In accordance with the numerous reports on the reduction of the risks that are associated with frozen‐thawed ET, the number of cases of frozen‐thawed ET that have been reported to the Society for Assisted Reproductive Technology has increased continuously, in comparison to the cases of fresh ET.^15^ Between 2007 and 2012, the number of frozen‐thawed ETs that was performed increased by 3.57‐fold, whereas the number of fresh ETs that was performed increased by 1.28‐fold. Although the increase in the rate of frozen‐thawed ETs was strikingly higher, this number exceeded the number of fresh ETs already being performed in 2008 in Japan, as depicted in Supplemental Figure 1B. As the technology of embryo cryopreservation continues to evolve, there is the potential for further increases in the frozen‐thawed ET rates.

### 4.2 Risk of a low birthweight with embryo transfer cycles

During fresh ET cycles, the ovarian stimulation procedures previously have been reported to show a significant correlation with the birthweight.^2^, ^16^, ^17^ The extended culture time of the blastocysts is known to decrease the risk of a low birthweight following a fresh ET cycle.^2^ The risk of a low birthweight following frozen‐thawed ET cycles is correlated with some factors, such as the patient's age, duration of embryo culture, luteal phase hormone therapies, sex of the neonate, and gestational age.^2^ Although the relative risk of a low birthweight increasing with maternal age also has been considered,^18^ the distribution of the maternal age in the present study was similar between these two ET methods (Figure 3B,D). Based on these results, the cause of the constant increase in the birthweight of singletons in the frozen‐thawed ETs cannot be explained. The mechanism needs to be elucidated in order to evaluate the clinical relevance and the safety of the ART treatments. In contrast, the mental and emotional impact of an earthquake had no effect on the risk of a lower birthweight (Figure 2).

### 4.3 Risk of Down syndrome with embryo transfer cycles

The risk of Down syndrome showed a significant difference between the two ET cycles from 2007‐2012 (Figure 3C, Table 1). The general frequency of Down syndrome at live birth is reported to be 39%^19^ and the frequency of Down syndrome in this study were between 13% and 19% (Table 1). Uninvasive prenatal genetic testing started in Japan in 2013. Before then, the optional Quadruple screen test and amniotic fluid test had been performed in general clinics in Japan. These prenatal screening tests might have lowered the frequency of Down syndrome in this study. The distribution of the maternal age and the birthweight were similar between the patients undergoing either method (Figure 3D). As the risk of Down syndrome increases with maternal age,^20^ this result raises the possibility that the use of frozen embryos might reduce the influence of maternal age. The registry data that were used in this study do not classify the date of embryo cryopreservation. The relationship between the maternal age at the time of embryo collection and the maternal age at the time of ET cannot be addressed. Importantly, these data do not indicate that frozen‐thawed ET is protective against Down syndrome.

In summary, in the present study, the impact of ART treatment was demonstrated on fetal growth and it showed that the neonatal birthweight is constantly increased with the frozen‐thawed ET method, compared to the fresh ET method. This result reveals the presence of a mechanism that affects the birthweight of neonates from frozen‐thawed ET cycles; however, this mechanism remains unknown. In order to assess the safety of the use of frozen‐thawed ET cycles, researchers should uncover this mechanism. The risk of Down syndrome also decreased with the frozen‐thawed ET method, compared to the fresh ET method. Further studies should be performed in order to fully understand the safety and efficacy of ART treatment.

## Disclosures

*Conflict of interest*: The authors declare no conflict of interest. *Human and Animal Rights*: This article does not contain any study with animal participants that have been performed by any of the authors.
