# Supplementary figures and images for: Birthweights and Down syndrome in neonates that were delivered after frozen‐thawed embryo transfer: The 2007‐2012 Japan Society of Obstetrics and Gynecology National Registry data in Japan
Source: Reprod Med Biol. 2017 Apr 10;16(2):228–34. doi: 10.1002/rmb2.12033 (PMC5661821; doi:10.1002/rmb2.12033)

Supplemental Fig.1

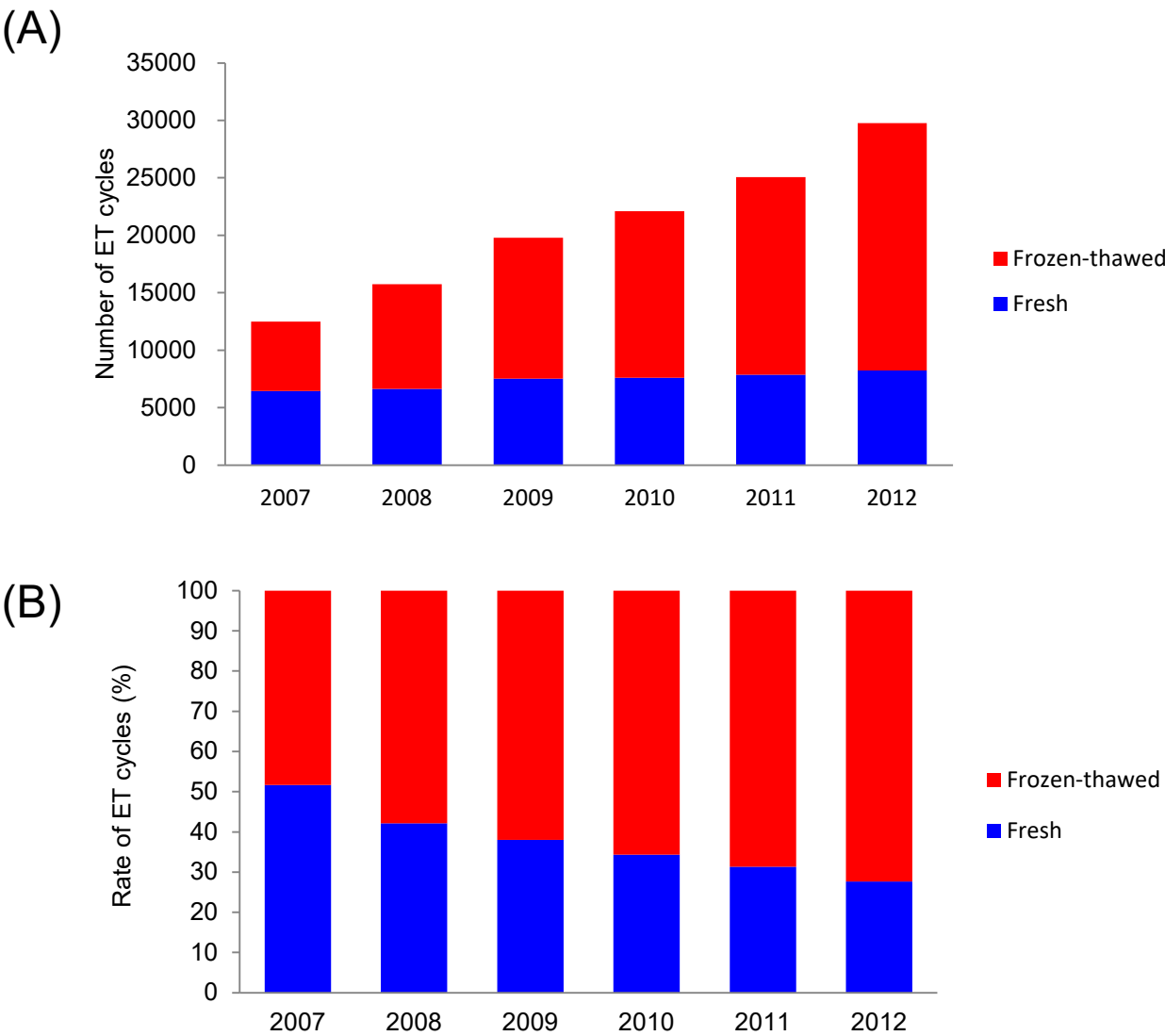

Supplemental Figure 1

Supplement: Supplementary file 1 [file RMB2-16-228-s001.pdf]

Supplemental Fig. 2

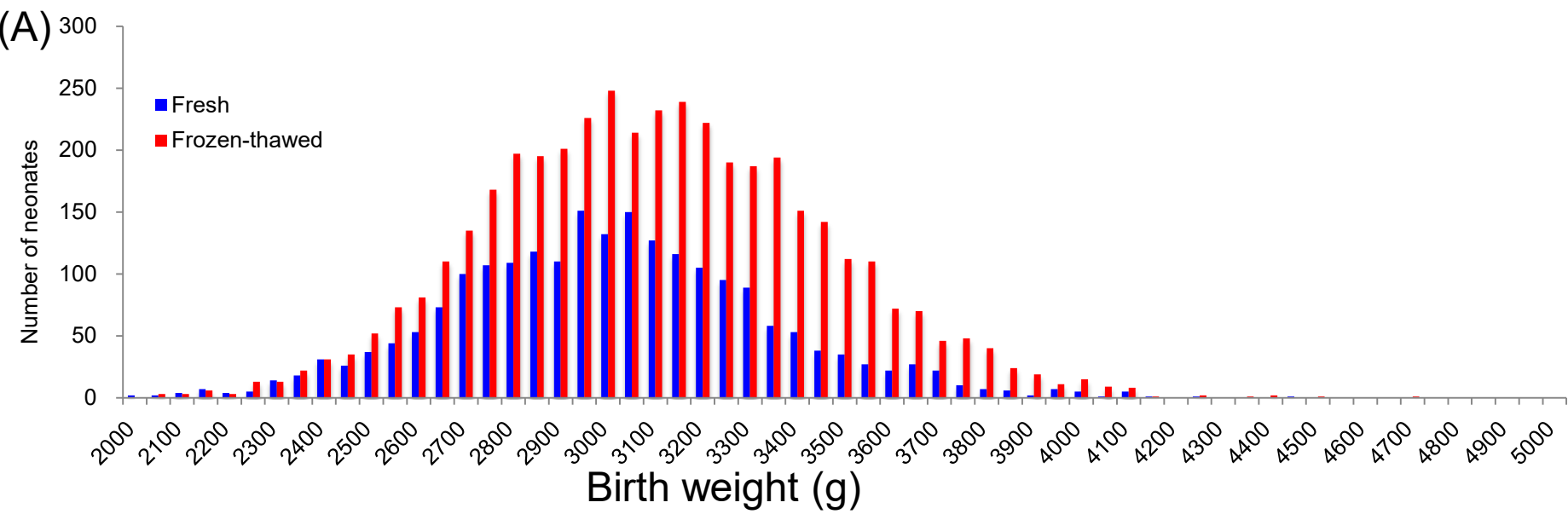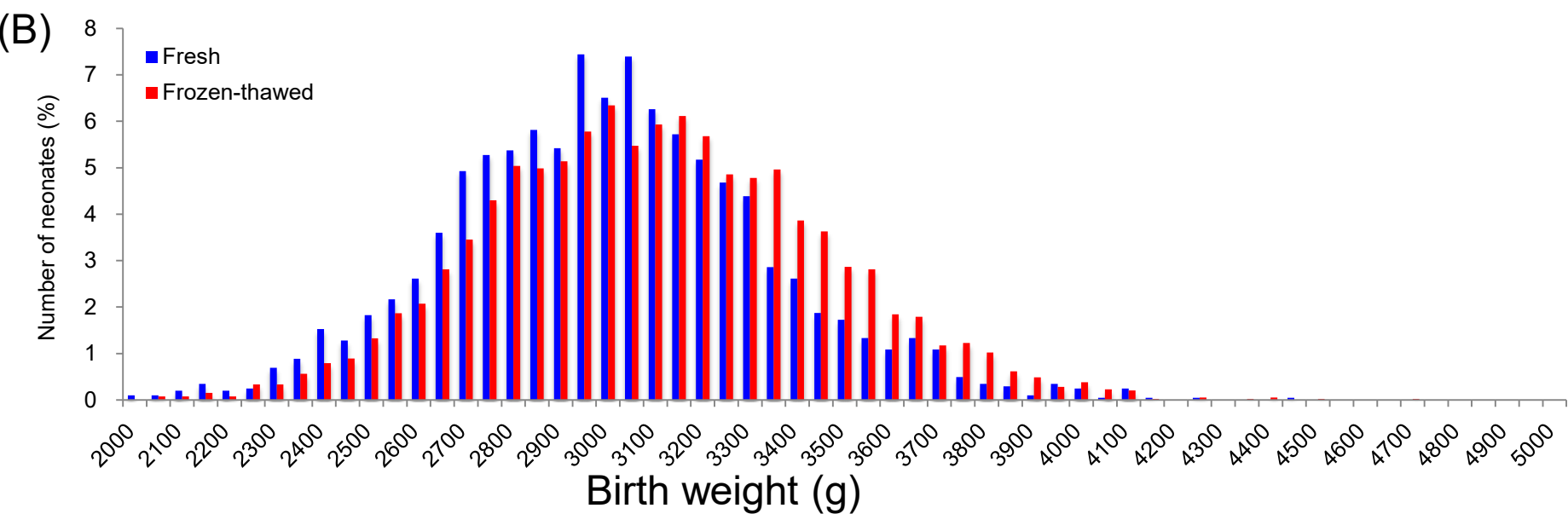

Supplemental Figure 2.

Supplement: Supplementary file 2 [file RMB2-16-228-s002.pdf]

Supplemental Fig. 3

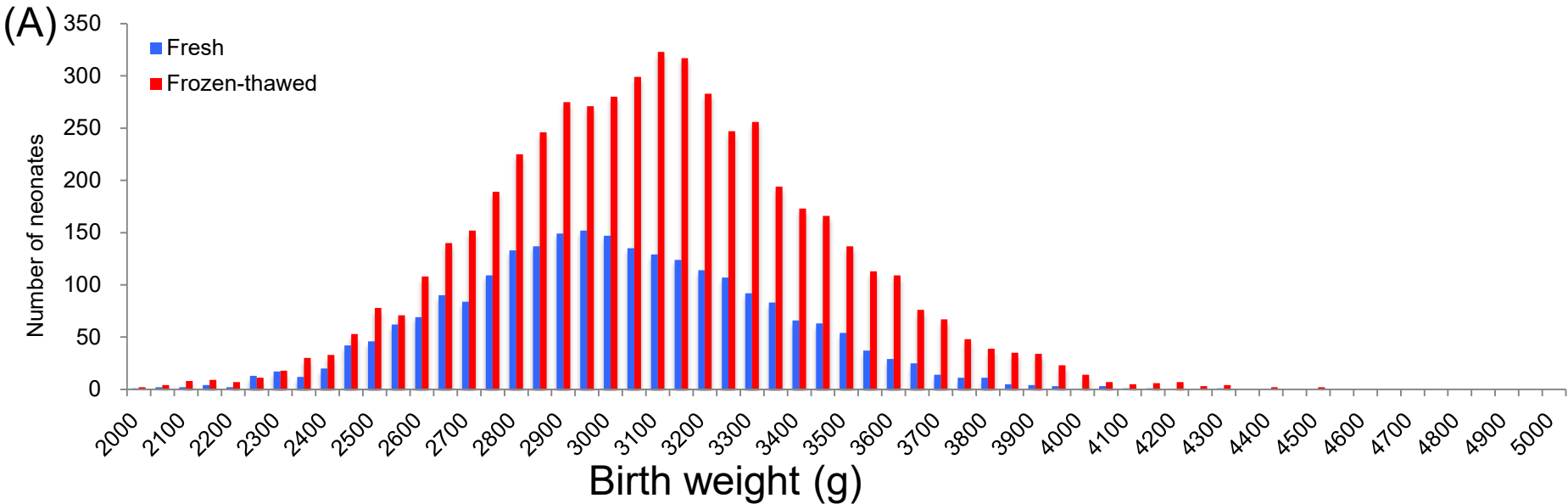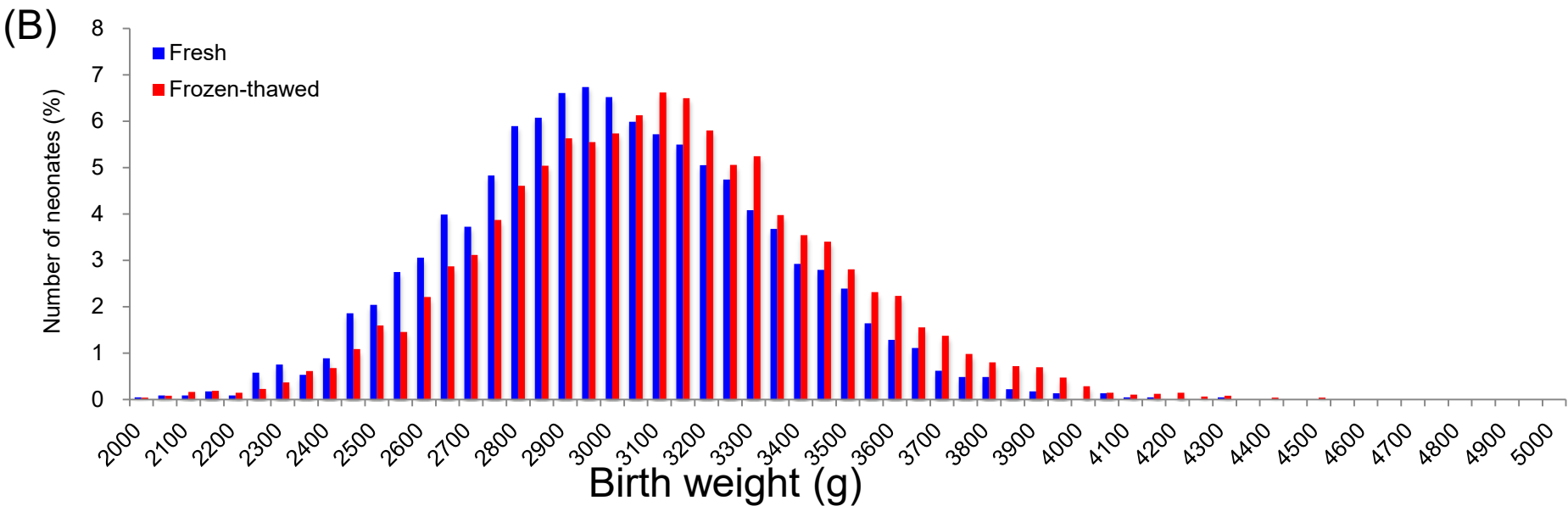

Supplemental Figure 3

Supplement: Supplementary file 3 [file RMB2-16-228-s003.pdf]
